# The Effect of Educational Intervention on Nurses' Attitudes and Beliefs about Depression in Heart Failure Patients

**DOI:** 10.1155/2014/257658

**Published:** 2014-11-26

**Authors:** Patricia Lea

**Affiliations:** University of Texas Medical Branch School of Nursing, 301 University Boulevard, Galveston, TX 77555-1029, USA

## Abstract

Systematic depression screening is feasible, efficient, and well accepted; however the lack of consistent assessment in heart failure inpatients suggests barriers preventing its effective diagnosis and treatment. This pilot study assessed the impact of an educational intervention on nurses' beliefs about depression and their likelihood of routinely screening heart failure patients. Registered nurses (*n* = 35) from adult medical-surgical units were surveyed before and after an educational intervention to assess their beliefs about depression prevalence and screening in heart failure patients. There was no significant influence on nurses' beliefs about depression, but the results suggested an increased likelihood that nurses would routinely screen for depression. The moderately significant correlation between beliefs and intent to screen for depression indicates that educational intervention could ultimately have a positive influence on patient outcomes through early detection and treatment of depression in patients with cardiovascular disease; however the observed increase in the intent to screen without a corresponding change in beliefs indicates other influences affecting nurses' intent to screen heart failure patients for depression.

## 1. Introduction

Over 4.9 million Americans suffer from heart failure, with 550,000 new cases diagnosed annually and new diagnoses expected to reach 1.5 million annually by the year 2040 [1]. The numerous symptoms associated with heart failure impair physical function, often causing social limitations, feelings of powerlessness, and inability to fulfill work and family roles. The resulting psychosocial stress can lead to frustration, disillusionment, and depression [2]. Three primary components influence the etiology of depression in heart failure patients: (1) symptomatology (e.g., fatigue, sleep problems); (2) behavioral characteristics (e.g., reduced physical activity); and (3) shared biological processes (e.g., neurohormonal inflammation and dysregulation). Polikandrioti et al. demonstrated a relationship between morbidity, hospital readmissions, and depression in heart failure patients, with major depression occurring four to five times more frequently among patients with heart failure than among the general population [1]. Significantly higher short- and long-term morbidity and mortality rates have been reported in patients with chronic heart failure suffering from major (but not minor) depression [3]. Depression in heart failure patients also contributes to decreased quality of life and inability to maintain activities of daily living [1], and is associated with a twofold greater risk for mortality, hospitalization, increased health care costs, and functional decline [4]. Although depression is a debilitating disease, it is frequently undiagnosed and untreated, with fewer than 50% of heart failure outpatients suffering from major depression receiving treatment [5].

Early identification of depression among heart failure patients is vital to providing timely treatment and limiting negative effects on patient outcomes [6]. Xiong et al. suggested that treatment of depression in heart failure patients could improve quality of life and social function, as well as health status [7]. Readmission rates three times higher have been found in patients with major depression as compared with those who have only mild or no depression [8]. Heart failure patients with depression have also been observed to experience longer hospital stays and received fewer cardiac procedures, components of heart failure education, and referrals to outpatient disease management programs [9]. These findings support the need for routine depression screening in heart failure patients [10].

The inpatient setting provides a valuable opportunity for depression assessment and treatment, allowing for daily monitoring of treatment side effects. The condition may otherwise progress unchecked for weeks or even months before the patient's next outpatient appointment [11]. If nurses routinely addressed the problem of depression in heart failure patients upon hospital admission, the length and severity of the heart failure episode could presumably decrease [12]. The commitment of nurses to the performance of routine screening for depression in heart failure patients is a function of their beliefs related to depression.

Previous studies have explored nurses' knowledge and practice with respect to routine depression screening in different patient populations. In a study performed by Zander that surveyed CNMs to assess their knowledge about postpartum depression and screening practices, the overwhelming majority of respondents acknowledged that the disorder is both serious and common but indicated that they do not currently perform regular screening due to lack of resources [13]. A 2012 study by de Man-Van Ginkel et al. found that nurses were able to use an established screening instrument, the patient health questionnaire (PHQ) 9, to effectively identify depression in recovering stroke patients who were able to complete the test [14]. Sanders also evaluated depression screening practices and factors associated with depression screening in a population of CNMs and observed a positive correlation of three factors associated with likelihood of screening for depression: attitude, perceived ability, and knowledge [15]. Ski and colleagues evaluated the impact of a training workshop on the implementation of a departmental screening policy and found that participating cardiac ward nurses were more aware of the prevalence of depression among cardiac patients and more confident about their ability to successfully identify patients at increased risk for depression [16]. Although Sowden et al. reported a positive response among cardiac care nurses to a departmental screening policy over a 12-month period, many hospitals do not include routine depression screening as part of their care protocols and the decision to screen is left to the discretion of individual nurses [17]. In the absence of established policy, patient outcomes may depend heavily on factors influencing the decisions of individual nurses about whether or not to screen for depression.

Worrall-Carter et al. conducted a study to identify nurses' knowledge and practice with respect to depression screening and follow-up referrals for patients with heart disease after the implementation of education workshops and deployment of a screening and referral tool [18]. They observed that a number of barriers hindered respondents' ability to screen for depression including limited knowledge, short length of hospital stay, and the development of depression over time causing difficulty in detecting symptoms during relatively brief periods of hospitalization [18]. These previous studies have suggested that depression screening alone or combined with small interventions (such as the provision of treatment algorithms) does not result in meaningful improvement of outcomes [19], indicating the presence of other barriers to the effective diagnosis and treatment of depression among inpatients suffering from heart failure.

Baldwin and Hirschfeld found that health professionals often misunderstand depression [20]. In a survey of general practitioners and practice nurses, Barley and colleagues observed that although the respondents identified depression as a problem among patients with heart disease, they were unsure of their roles in managing it and did not know how to achieve an individualized treatment approach [21]. Successful recognition of depression in heart failure patients requires that nurses understand the importance of assessment, be capable of assessing patients for depression, and possess the necessary tools to conduct effective assessments. Simple awareness of depression and available assessment tools is not sufficient to make nurses engage in routine screening; they must choose to incorporate depression screening into their clinical practice. Haws et al. surveyed 707 nurses and 106 general practitioners about their attitudes and beliefs with respect to depression after myocardial infarction [22]. They found that practitioners who had received training had more positive attitudes toward screening for depression and felt more confident about their ability to recognize the symptoms of depression in patients recovering from myocardial infarction.

According to Conner and Armitage, highly motivated individuals think deliberately and plan their behavior based on their attitudes and beliefs [23]. Nurses lacking motivation or having little support for evidence-based practice changes are often unsuccessful in implementing behavioral changes, suggesting that nurses' attitudes and beliefs strongly predict behavioral outcomes [24].

In order to produce a positive practice change, it is important to study nurses' beliefs regarding depression and screening to assess whether these beliefs are predictive of their behavior. Assessment of nurses' beliefs and attitudes will provide evidence to inform positive behavioral influences that increase their probability of performing depression screenings. Nurses who use a valid and reliable depression assessment tool can improve their recognition of depression, and ultimately treatment outcomes, in heart failure patients. This study evaluated whether an educational intervention to better inform beliefs about depression and the use of screening in heart failure patients would increase the likelihood that nurses would use a validated screening tool to routinely assess for depression.

## 2. Methods

### 2.1. Research Design

A descriptive research design was used to assess nurses' beliefs and attitudes regarding depression prevalence and screening in heart failure patients. This quantitative study used a one-group pretest, posttest, intervention design to assess nurses' beliefs and attitudes related to depression prevalence and screening in heart failure patients. The approach involved administration of an identical group pretest, educational intervention, and posttest (01—pretest, X—educational intervention, and 02—posttest).

### 2.2. Sample

The study population included a purposive sample of 206 registered nurses working full time, part time, and PRN (per diem) on adult medical-surgical units at a large urban teaching hospital in southeast Texas. Exclusion criteria included licensed vocational nurses and agency nurses. It was also required that participants be capable of reading English and using a computer. A power analysis was completed to estimate the minimum sample size necessary to obtain significant results. To produce a standardized size effect, the effect size in which the outcome variable was divided by the standard deviation (*d* = 0.5), a minimum sample size of 34 participants was needed.

### 2.3. Research Instrument

Demographic information included six questions about gender, age, years of RN experience, current employment status (i.e., full time, part time, PRN, and agency), years with current employer, and highest degree attained (i.e., associate, bachelor, master, and doctorate). Data were collected using the Revised Depression Attitude Questionnaire (DAQ), with the permission of Dr. Mark Haddad (M. Haddad, personal communication, June 20, 2013). Minor revisions were made to improve ease of understanding because the questionnaire was written using British English; however the changes did not alter its reliability or validity. The questions included in the DAQ were related to

- comfort level in dealing with physical versus mental illnesses, and specifically in dealing with the needs of depressed patients;
- confidence in assessing and responding to suicide risk in depressed patients;
- beliefs about the potential for successful treatment, as well as the potential effectiveness of antidepressant or psychological therapies or other treatments;
- belief that anyone can suffer from depression versus the condition being a natural part of aging or adolescence;
- attitude toward caring for depressed patients and about the role of the nursing profession in general in addressing depression;
- belief that depression is a medical condition requiring treatment versus transient unhappiness or a lack of self-discipline or willpower;
- beliefs about who is responsible for recognizing and managing depression (i.e., nurses or other medical staff, or both);
- beliefs about the relationship between depression and heart failure.

The original DAQ was developed by Botega et al. and measured doctors' attitudes regarding depression [25]. Continuing research suggests that the DAQ remains a useful instrument to guide educational efforts and measure change over time [26]. Dr. Haddad later revised the instrument to include nurses in his studies of depression. The revised questionnaire consists of 20 questions eliciting responses according to a five-point Likert scale format representing answers ranging from strongly agree to strongly disagree. Reliability and internal consistency improved compared with the original DAQ, with a Cronbach's alpha of 0.85 for the total revised scale.

The 20 questions in the Revised Depression Attitude Questionnaire also demonstrated face, content, and some elements of construct validity. To demonstrate face validity, questions 21–25 were emailed to three Ph.D. prepared senior nurse researchers, the Director of Research at the institution in which the study was conducted, and nine members of the institution's Research Council for review. The questions were rated on a scale of 0–4 (0 = not valid to 4 = valid) and all responses ranged from 3 to 4.

Five questions were added to this instrument to capture additional information about attitudes, specifically those related to screening, that were not covered in the original instrument. The additional questions assessed

- beliefs about the need for depression screening in heart failure patients;
- confidence in screening for depression;
- perception of whether workload limits depression screening; and
- performance of screening upon hospital admission.

### 2.4. Data Collection

The research proposal was approved by the Institutional Review Boards (IRBs) at the university where the study was conducted and the researcher's affiliated institution before data collection was initiated. Only aggregate data was reported, and all data was stored on a password-protected computer accessible only to the researcher to protect participant privacy. Data was collected over a four-week period using SurveyMonkey.com, a web-based program that administers the survey and compiles the responses. SurveyMonkey.com requires participants to create a unique username and password that must be entered each time a user logs into the website. In addition, the site protects user information through Secure Sockets Layer (SSL) technology using both server authentication and data encryption, ensuring that user data remains secure and is available only to authorized persons. The Director of Education at the hospital where the study was conducted assisted the researcher in setting up the study materials (the pretest, educational intervention, and posttest) with a link to permit access only to the desired participants.

### 2.5. Electronic Survey Questionnaire

After reading the study informed consent letter, participants were directed to indicate their agreement to participate in the study and were then automatically directed to the demographic questions. After providing the requested demographic data, participants continued to the study pretest, the educational intervention, and the study posttest.

### 2.6. Educational Intervention

A web-based tutorial was developed as the educational intervention. This method was used in order to accommodate scheduling constraints of the sample, allowing the nurses to access the survey and intervention independently, online, and according to their own availability. After consenting to participate, nurses were directed to complete the pretest, view the educational intervention (a PowerPoint slide presentation), and complete the posttest. Completion of the tutorial was required in order for participants to access the posttest; therefore completion of the posttest provided verification that the presentation was viewed. The objectives of the educational intervention were to (a) describe the emotional and physical issues associated with depression; (b) define the American Heart Association's recommendation for depression screening; (c) explore the signs and symptoms of depression in heart failure patients; and (d) understand the importance of depression screening. The presentation included 17 slides covering the definition and symptoms of depression, the relevance of depression in the management of heart failure patients, and some erroneous beliefs that may negatively influence nurses' likelihood of routinely screening for depression in heart failure patients.

### 2.7. Statistical Analysis

The raw data collected from SurveyMonkey.com was transferred to an Excel spreadsheet, which was then coded and transferred to the Statistical Package for Social Sciences program (SPSS) version 20.0, (2012, Chicago, IL) for analysis. The data was cleaned to eliminate any entry errors. Polit and Beck state that data entry is prone to error and that it is essential to verify entries and correct mistakes [27]. One data cleaning method involved visually comparing the numbers on a printout of the data file with codes on the original source. Even verified data usually contains some errors from coding problems or misreported information. Another method of cleaning is to check for outliers, which are values that lie outside the normal range of values. The data was visually inspected for any outliers in the variable data set. The researcher noted that one of the variables, age, had a data entry of 4 years. Because this was not valid response for the target population, this entry was corrected.

To test the internal consistency of the items, Cronbach's alpha values were calculated. Tavakol and Dennick suggest that Cronbach's alpha is an important concept in the evaluation of assessments and questionnaires and that assessors and researchers must estimate this quantity to add validity and accuracy to the data interpretation [28]. Alpha was calculated for each of the 20 questions in the original questionnaire and for the five additional questions regarding intent to screen. A maximum alpha score of 0.90 has been recommended because high alpha values (>0.90) may suggest redundancies indicating that the test length should be reduced [29]. The data was also evaluated for normality and homogeneity using a parametric test if the distribution was normal or a nonparametric test if the distribution was not normal.

Descriptive statistics (means, medians, and standard deviations) were calculated to define the population and identify trends among study variables. A paired *t*-test was used because of the pretest/posttest modality; however, an analysis of the homogenous variances—the *F* test—was conducted to determine the type of variance used for the paired *t*-test, a null hypothesis H(0) if the variances are homogeneous, and H(1) if the variances are not homogenous. The *F* value was 0.3849 and the *P* value was 0.00, with df = 34. Because of the heterogeneity of the variance, a paired *t*-test with unequal variances was used.

## 3. Results

### 3.1. Demographics

The pretest, educational intervention, and posttest were emailed to 206 registered nurses working full time, part time, or per diem in designated adult medical surgical units at a large urban hospital. A total of 56 nurses responded to the study. Of those participants, 21 were rejected due to incomplete information, including 12 subjects who did not complete the posttest, 1 subject who did not meet the established inclusion criteria (agency nurse), 3 subjects who chose not to participate, and 3 subjects who completed the posttest but not the pretest. Using listwise deletion, which is a method that deletes entire observations if there are missing values, two entire observations were deleted. Polit and Beck state that this method is the simplest to use with missing information [27]. The survey yielded 35 eligible subjects, for a response rate of 16.9%. Demographic data characterizing the sample and identifying trends among study variables is included in Table 1.

### 3.2. Psychometric Properties of the Adapted Questionnaire

The test items (pre and post) were scored based on the mean values of the Likert scale responses. To ensure that the scale was reliable and valid, Cronbach's alpha values were calculated for the original questionnaire (Revised Depression Attitude Questionnaire) and the five questions that were added to an already valid and reliable questionnaire. Internal consistency describes the extent to which all items in a test measure the same concept or construct (Table 2). Number of test items, item interrelatedness, and dimensionality affect the value of alpha, for which an acceptable range of 0.70–0.95 has been proposed. Although the alpha values for the 25 questions in the DAQ were in this range, values for the five additional questions were 0.50 for premean test scores and 0.67 for postmean test scores. The premean alpha score was low because the questions were added and had not been rigorously tested; however, the primary research question was concerned with the postmean measures of intent to screen, which did produce a higher Cronbach's alpha than the premean scores. The low Cronbach's alpha was expected because the questions had not been used previously and the number of questions was low. The postmean test score was most important and had a better Cronbach's alpha score, as it most directly addressed the primary research question.

The Shapiro-Wilk test was used to demonstrate normality of the data, where the value of *W* lies between zero and one. Small values of *W* result in the rejection of normality, whereas a value of one indicates normality of the data. As indicated in Table 2, the calculated premean *W* value is 0.94 and the postmean *W* is 0.94—revealing scores closer to one and suggesting a normal distribution of both groups (before and after the educational intervention).

### 3.3. Effectiveness of Educational Intervention

Data analysis was conducted to assess nurses' beliefs regarding depression before and after the educational intervention. Lower scores on the Likert scale indicated more positive beliefs regarding depression. The paired *t*-test was performed to determine whether scores increased as a result of an educational intervention. The results suggested that nurses developed stronger beliefs about depression after the educational intervention. With a *P* value of <0.05, the calculated values *t* = 1.74 and *P* = 0.09 indicated no significant difference between the results before and after the educational intervention. The demographic data support this finding, in that the majority of subjects were of age 40–59 (*n* = 21) and it could be that their foundational beliefs related to depression were already well established. Based on the pre- and postmean test scores, it is reasonable to conclude that the educational intervention did not substantially affect nurses' beliefs related to depression. For the purposes of this study, intent to screen was defined as the state of the nurses' mind that directs him or her toward a specific goal (screening for depression in heart failure patients). As shown in Table 3, the scores measuring beliefs related specifically to intent to screen were lower after the educational intervention, suggesting a greater intent to screen.

A Pearson *r* correlation was performed between the premean and postmean scores to detect any association between beliefs and intent to screen patients. The results (Table 4) demonstrate a moderately significant correlation between the premean and postmean scores. Moreover, the postmean score increased after the educational intervention as compared with before, which suggests a corresponding increase in intent to screen intent to screen were lower after the educational intervention, even though there was no significant corresponding change in the nurses' beliefs.

## 4. Discussion

No data has been previously published linking nurses' beliefs to their intent to screen for depression in heart failure patients. Butler and Kalogeropoulos reported that hospitalizations account for the majority of the $39 billion spent annually for heart failure care [30]. With heart failure readmissions estimated to cost the American public more than $15 billion per year, reducing hospital readmission rates is a national priority. It is not surprising that heart failure hospitalizations are a focal point for quality improvement and cost reduction attempts in the face of shrinking health care funding and the elimination of reimbursements for 30-day heart failure readmissions as of October 2012. Although the data presented here did not indicate a significant change in the beliefs of the participating nurses with respect to depression before and after the educational intervention, the findings did suggest a moderately significant increase in intent to screen. Despite the moderately significant correlation between beliefs and intent to screen for depression, the intent to screen improved without a corresponding change in beliefs, suggesting that factors other than beliefs influence the likelihood of screening.

The findings of Poštuvan et al. contradict the data presented in this study, stating that the low identification rate of depression might result from nurses' lack of knowledge and negative attitudes [31]. Although the sample population was relatively small, the current study indicates that nurses' beliefs and attitudes are generally positive and that even minimal education can produce an increase in the intent to screen. Nursing leaders, at the forefront of efforts to prevent heart failure readmissions, must understand that if nurses truly believe in the value of a desired behavior, their intent to perform the behavior increases. The impact of depression screening on patient outcomes and health care costs create a powerful incentive to better educate nurses on the importance of screening in vulnerable populations such as heart failure patients. This study supports the positive correlation between nurses' beliefs and their intent to screen; however, the increase in intent to screen without a corresponding change in the nurses' beliefs indicates the influence of other factors.

*Recommendations for Future Research*. Because the web-based educational intervention used in this study (PowerPoint presentation) demonstrated minimal significant effect, one recommendation would be to implement face-to-face educational workshops that address the issue of depression in heart failure. Nurses' attendance of education workshops could contribute to an improvement of their beliefs and attitudes toward depression, resulting in greater commitment to screening. Other future research might include focus groups with nurses concerning depression screening in heart failure patients or exploration of new methods that can be used by nurses to screen for depression in heart failure patients (use of questionnaires, etc.).

One limitation of this pilot study was the low response rate (35 subjects of the 206 potential subjects). Because of the small sample, the results observed in this study may not be globally applicable across the general nursing population. Future studies with larger sample populations of nurses from different hospitals and different geographic areas would incorporate potential cultural variances regarding beliefs about depression. In addition, the pre/post design did not include a control group, which should be added in future studies to allow for assessment of any baseline changes in responses to the questionnaire that cannot be attributed to the educational intervention. Another limitation of the study is that potential correlations between demographics and outcomes were not explored. Additional research could also explore correlations of age, gender, and years of experience with nurses' beliefs about depression and intent to screen.

Knowledge was not directly assessed in this study because the scaled responses in the DAQ were intended to measure beliefs and attitudes and were not appropriate to measure knowledge. In communications with Dr. Haddad, who revised the DAQ, he stated that attempts to measure knowledge and beliefs together should be cautious. Future studies might include measurement of nurses' knowledge about depression and screening in heart failure patients. Another limitation of the study is that potential correlations between demographics and outcomes were not explored, which might also provide a basis for future studies.

Although it has been shown that nurses can successfully identify cardiovascular patients at risk for depression using available screening instruments [32], the ability of these instruments to effect positive patient outcomes depends greatly on when and how nurses use them. The results of this study demonstrate that even minimal education can influence the likelihood that nurses will routinely screen heart patients for depression.

## Figures and Tables

**Table 1:** Demographic information (*n* = 35).

Characteristic	*N*	Percentage
Gender		
Male	6	17.1%
Female	29	82.9%
Age (years)		
20–29	2	5.7%
30–39	6	17.1%
40–49	10	28.5%
50–59	11	31.4%
60–70	6	17.1%
	Mean 48.3	SD 10.8%
Years of experience		
0–9	12	34.3%
10–19	11	31.4%
20–29	7	18.4%
30–39	4	10.5%
40–49	1	2.6%
	Mean 15.6	SD 11.21%
Employment status		
Full time	33	94.0%
Part time	2	5.6%
Per diem	0	0.0%
Highest degree		
Diploma	1	2.8%
Associate	13	37.1%
Bachelor	17	48.6%
Master	3	8.6%
Doctorate	1	2.8%

**Table 2:** Cronbach's alpha for the DAQ and five added questions.

	Pre	Post
All 25 questions	0.81	0.84
Original DAQ (20 questions)	0.80	0.82
Screening subscale (5 questions)	0.50	0.67

**Table 3:** Screening items.

Pre	
Mean	2.41
SD	0.59
*P*	0.01
Post	
Mean	2.07
SD	0.65
*P*	0.00

**Table 4:** Correlation table.

Pre	Post
*r* = 0.42	*r* = 0.51
*P* = 0.01	*P* = 0.00
